# Association of Neuropathologically Confirmed Frontotemporal Dementia and Alzheimer Disease With Criminal and Socially Inappropriate Behavior in a Swedish Cohort

**DOI:** 10.1001/jamanetworkopen.2019.0261

**Published:** 2019-03-29

**Authors:** Madeleine Liljegren, Maria Landqvist Waldö, Alexander Frizell Santillo, Susann Ullén, Robert Rydbeck, Bruce Miller, Elisabet Englund

**Affiliations:** 1 Division of Oncology and Pathology, Department of Clinical Sciences, Lund University, Region Skåne, Sweden; 2 Department of Genetics and Pathology, Medical Service, Region Skåne, Sweden; 3 Division of Clinical Sciences, Helsingborg, Department of Clinical Sciences, Lund University, Sweden; 4 Clinical Memory Research Unit, Department of Clinical Sciences, Lund University, Lund, Sweden; 5 Clinical Studies Sweden–Forum South, Skåne University Hospital, Lund, Sweden; 6 Department of Forensic Psychiatry, Trelleborg, Region Skåne, Sweden; 7 Memory and Aging Center, Department of Neurology, University of California, San Francisco

## Abstract

**Importance:**

Criminal and socially inappropriate behavior is encountered among patients with dementia, and it is sometimes the first sign of a dementing disorder. This behavior constitutes a significant burden to society, patients’ relatives, and patients themselves.

**Objectives:**

To investigate and compare the prevalence and type of criminal and socially inappropriate behavior, as well as recurrence of criminal behavior, associated with Alzheimer disease (AD) and frontotemporal dementia (FTD) neuropathologically verified post mortem, and to assess whether there is a specific type of protein pathology more closely associated with criminal behavior in patients with FTD.

**Design, Setting, and Participants:**

Cohort study using medical record review of 220 Swedish patients with a postmortem neuropathologic diagnosis of AD (n = 101) or frontotemporal lobar degeneration (n = 119) (hereinafter referred to as FTD) diagnosed between January 1, 1967, and December 31, 2017.

**Main Outcomes and Measures:**

Patient notes containing reports of criminal and socially inappropriate behavior, as well as data on dominant protein pathology for patients with FTD, were duly reviewed and recorded. The Fisher exact test or logistic regression was used to assess possible differences between groups.

**Results:**

Of the 220 patients studied, 128 (58.2%) were female, the median (range) age at disease onset was 63 (30-88) years and at death was 72 (34-96) years, and the median (range) disease duration was 9 (1-28) years. Instances of criminal behavior were found in 65 of the 220 patients (29.5%): in 15 of the 101 patients (14.9%) with AD and 50 of the 119 patients (42.0%) with FTD (*P* < .001). Recurrence of criminal behavior was significantly higher in the FTD group (89.0%) than in the AD group (53.3%) (*P* = .04). Instances of socially inappropriate behavior were found in 57 patients (56.4%) with AD and 89 (74.8%) with FTD (*P* = .004). An expression of non-tau pathology increased the odds for criminal behavior by a factor of 9.0 (95% CI, 3.4-24.0) among patients with FTD.

**Conclusions and Relevance:**

These results suggest that criminal and socially inappropriate behaviors may be more prevalent and criminal behaviors may be more recurrent in patients with FTD than in those with AD. Non-tau pathology, but not tau pathology, appears to be associated with criminal behavior. These findings may help with the clinical diagnostic process.

## Key Points

**Question:**

Is criminal and socially inappropriate behavior more common among patients with frontotemporal dementia than in those with Alzheimer disease, and is a certain type of protein pathology associated with criminal behavior in patients with frontotemporal dementia?

**Findings:**

This cohort study of 220 Swedish patients with neuropathologically verified frontotemporal dementia or Alzheimer disease found a significantly higher prevalence of criminal and socially inappropriate behavior among those with frontotemporal dementia compared with those with Alzheimer disease. An expression of non-tau pathology significantly increased the odds for criminal behavior among patients with frontotemporal dementia.

**Meaning:**

These findings might help in the clinical diagnostic process, especially when the clinical picture is unclear and the differential diagnoses are difficult to distinguish.

## Teaser

This cohort study reviews medical records to compare the prevalence of criminal and socially inappropriate behavior between Swedish patients with Alzheimer disease and those with frontotemporal dementia confirmed neuropathologically and investigates the association of protein pathology with criminal behavior in the frontotemporal dementia group.

## Introduction

Previous research has shown that criminal behavior and socially inappropriate behavior are encountered among patients with dementia; in fact, they are sometimes the first sign of a dementing disorder.^1,2^ Criminal behavior ranges from violence to sexual advances, theft, and traffic violations.^3,4,5,6,7^ Socially inappropriate behavior may manifest as impulsive and disinhibited action, including open talk about private matters, rude comments to others, and maladaptive emotional reactions.^8^ In general, neurobiological explanations for criminal and socially inappropriate behavior use a range of models, from structural to neurochemical. In the context of neurodegenerative diseases, deviant behaviors are often associated with damage to the frontotemporal areas.^8,9,10^ Criminal and socially inappropriate behaviors constitute a significant burden to society, patients’ relatives, and patients themselves; they may result in substantial financial loss and caregiver distress.^11,12,13,14^

A recent study showed that patients with frontotemporal dementia (FTD) interact more frequently with the police than patients with Alzheimer disease (AD) and that the interaction is most often because of criminal behavior.^15^ The first aim of the present study was to investigate and compare the prevalence, recurrence, and type of criminal behavior and prevalence and type of socially inappropriate behavior among patients diagnosed with dementia that was neuropathologically verified at postmortem examination as AD or frontotemporal lobar degeneration (FTLD).^16,17,18,19,20^ The term FTD is hereinafter used for the neuropathologic term FTLD, encompassing a group of brain diseases that correlate to the following clinical diagnoses: behavioral variant of frontotemporal dementia, semantic dementia/semantic variant of primary progressive aphasia, progressive nonfluent aphasia, corticobasal degeneration/corticobasal syndromes, and progressive supranuclear palsy.^19,20,21,22,23^ The second aim was to study the time of occurrence of criminal behavior in these patients. The third aim was to investigate whether, within the FTLD group, there is a certain type of protein pathology more closely associated with criminal behavior than other types.

## Methods

We considered, from a cohort of neuropathologically diagnosed individuals, 119 patients with a diagnosis of disease within the FTLD spectrum and 101 patients with a diagnosis of AD. Tissue from diagnosed individuals was retrieved from the brain bank at the Department of Genetics and Pathology, Medical Service, Lund, Region Skåne, Sweden. Our analysis included an extensive postmortem examination of cases recorded between January 1, 1967, and December 31, 2017; this patient cohort has been described in a previous study.^7^ We added 22 additional cases of FTLD to the 97 cases considered in the previous study. All patients had earlier been referred to specialists in geriatric psychiatry or cognitive medicine at the Memory Clinic (previously the Psychogeriatric Department) in Lund and closely monitored during the entire course of disease, as recorded in longitudinal research studies. In Sweden, ethical laws do not apply to decedents; therefore, there was no need for institutional review board approval. However, to be certain that our study conformed to ethical guidelines, we applied to the Regional Ethical Review Board in Lund, which issued a favorable judgment, declaring that there were no ethical contradictions in the study. This study followed the guidelines established by the ethical review board.

The predominant (and generally sole) protein pathology (tau, transactive response DNA-binding protein 43, fused in sarcoma, or other) from the neuropathology reports of the patients with FTLD was noted. Patient demographic characteristics are presented in Table 1. All medical records available for the 220 patients (including referral letters, biochemical analyses, and copies of radiology findings from various clinical investigations) were reviewed in search of clinical data on demographic characteristics, behavioral disturbances, and other issues. Reviewers were not blinded to the neuropathologic diagnoses. Patient notes containing reports of criminal and socially inappropriate behavior were reviewed and noted. Prevalence of criminal and socially inappropriate behavior among patients was determined.

**Table 1.** Demographic Characteristics of the 220 Study Patients With Dementia

Characteristic	Patient Group by Diagnosis		
	All (N = 220)	AD (n = 101)	FTD (n = 119)
Sex, No. (%)			
Male	92 (41.8)	34 (33.7)	58 (48.7)
Female	128 (58.2)	67 (66.3)	61 (51.3)
Age at disease onset, median (range), y	63 (30-88)	64 (44-88)	60 (30-84)
Age at death, median (range), y	72 (34-96)	76 (57-96)	70 (34-94)
Disease duration, median (range), y	9 (1-28)	10 (1-23)	8 (1-28)

Abbreviations: AD, Alzheimer disease; FTD, frontotemporal dementia.

Criminal behavior encompasses acts that violate the law^24^ as well as those that deviate from traditional social decorum and could potentially lead to legal ramifications.^5^ Socially inappropriate behavior encompasses acts that deviate from traditional social decorum or the patient’s personality but would not lead to legal ramifications.

Details about the recurrence of criminal behavior were categorized according to the number of incidents (1 or >1). Types of criminal behavior and socially inappropriate behavior were mapped and classified into the following categories: mismanagement of personal finances, public urination or defecation, sexual advances, theft, traffic violations, and other. We also studied the prevalence of patient interaction with police and its cause (ie, whether or not it was associated with criminal behavior). We evaluated whether patients’ behavior within each category reached criminal levels or was merely socially inappropriate. We chose not to assess physical aggression toward other persons or living creatures when mapping criminal and socially inappropriate behavior because this particular phenomenon has already been examined in a previous study.^7^

Times of onset of criminal behavior during the course of disease (ie, during its first or second half) were noted. We then compared findings on criminal behavior with data on the predominant protein pathology for the FTD group.

### Statistical Analysis

Demographic data were described numerically, in terms of percentages or medians with minimum and maximum values. The Fisher exact test or logistic regression was used to assess possible differences between groups, and 2-sided *P* < .05 was considered statistically significant. Statistical analyses were performed using SPSS Statistics 25 (IBM Corp).

## Results

### Quantitative Results

#### Criminal Behavior

Of the 220 patients studied, 128 (58.2%) were female, the median (range) age at disease onset was 63 (30-88) years and at death was 72 (34-96) years, and the median (range) disease duration was 9 (1-28) years. Sixty-five patients (29.5%) exhibited behavior that could be considered criminal during the course of disease. The distribution of criminal behavior was 15 incidents among the 101 patients (14.9%) in the AD group and 50 among the 119 (42.0%) in the FTD group, yielding a statistically significant difference between the 2 groups (*P* < .001) (Table 2). After adjusting for age and sex, the difference was still significant (*P* < .001), with an odds ratio of 3.5 (95% CI, 1.8-7.1). There were 10 patients with AD who committed 1 type of crime, 5 who committed 2 or 3 different types of crime, and none who committed more than 3 different types of crime. Corresponding numbers for patients with FTD were 30 for 1 type, 18 for 2 or 3 types, and 2 for more than 3 types of crime. Recurrence of criminal behavior also differed between diagnostic groups: of the 15 patients with AD who exhibited criminal behavior, 7 (46.7%) committed a crime once and 8 (53.3%) did so more than once. Corresponding numbers for the 50 patients with FTD and criminal behavior were 9 (18.0%) with 1 incident of criminal behavior and 41 (82.0%) with more than 1, again yielding a statistically significant difference (*P* = .04).

**Table 2.** Distribution of Different Types of Behavior Within Patient Groups

Behavior	No. (%)		*P* Value
	AD (n = 101)	FTD (n = 119)	
Criminal behavior			
Mismanagement of personal finances	0	8 (6.7)	.008
Public urination or defecation	0	1 (0.8)	>.99
Sexual advances	2 (2.0)	11 (9.2)	.04
Theft	2 (2.0)	16 (13.4)	.002
Traffic violations	2 (2.0)	22 (18.5)	<.001
Other^a^	13 (12.9)	26 (21.8)	.11
Socially inappropriate behavior			
Mismanagement of personal finances	1 (1.0)	29 (24.4)	<.001
Public urination or defecation	30 (29.7)	21 (17.6)	.04
Sexual advances	5 (5.0)	22 (18.5)	.003
Traffic violations	7 (6.9)	25 (21.0)	.004
Other^b^	48 (47.5)	84 (70.6)	.001

Abbreviations: AD, Alzheimer disease; FTD, frontotemporal dementia.

^a^ Other criminal behavior included threats, vandalism, pyromania, and stalking.

^b^ Other socially inappropriate behavior included aimless screaming, crying, or laughing.

Table 2 details the types of crime and their distribution within each group. Criminal behavior in the “other” category ranged from verbal (including homicidal) threats to vandalism, pyromania, and stalking.

Nine of the 101 patients (8.9%) with AD interacted with the police compared with 30 of the 119 patients (25.2%) with FTD, yielding a statistically significant difference between groups (*P* = .002). Furthermore, 18 of the 30 patients (60.0%) with FTD did so owing to criminal behavior, whereas that was applicable to only 2 of the 9 patients (22.2%) with AD; the difference between groups was not statistically significant in this case (*P* = .07). Overall, 18 of the 119 patients (15.1%) with FTD interacted with the police because of criminal behavior, whereas this was the case for only 2 of the 101 patients (2.0%) with AD, a statistically significant difference between both groups (*P* = .001).

In the 119 patients with FTD, we found 22 (18.5%) to have had some kind of note about psychiatric history before symptom onset. Of the 50 patients with FTD who exhibited criminal behavior, 9 (18.0%) had experienced psychiatric problems before symptom onset and had been diagnosed with 1 of the following disorders: depression (5 patients), alcohol abuse (3), and borderline personality disorder (1).

#### Socially Inappropriate Behavior

A total of 57 of the 101 patients (56.4%) with AD and 89 of the 119 patients (74.8%) with FTD exhibited socially inappropriate behavior during the course of disease, again yielding a statistically significant difference between patient groups (*P* = .004). After adjusting for age and sex, the difference was still significant (*P* = .01) with an odds ratio of 2.2 (95% CI, 1.2-4.1). The Figure provides a graphic comparison of criminal behavior and socially inappropriate behavior in both groups. Patients with FTD exhibited a greater prevalence of socially inappropriate behavior in every category under study except for public urination or defecation, in which patients with AD dominated (30 vs 21; Table 2). Most patients who exhibited criminal behavior also exhibited socially inappropriate behavior (13 of 15 patients with AD [86.7%] and 47 of 50 patients with FTD [94.0%]).

**Figure.**
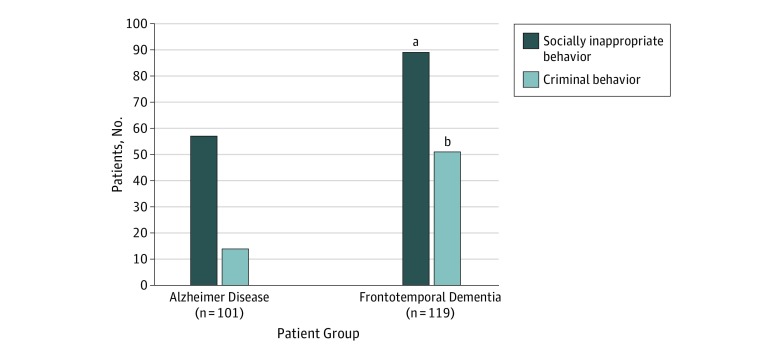
Criminal and Socially Inappropriate Behavior in Patients With Alzheimer Disease and Frontotemporal Dementia ^a^*P* = .004 compared with patients with Alzheimer disease. ^b^*P* < .001 compared with patients with Alzheimer disease.

The time of onset of criminal behavior was evenly spread between groups. Almost half the patients exhibiting criminal behavior (7 of the 15 patients [46.7%] with AD and 23 of the 50 patients [46.0%] with FTD) did so for the first time during the first half of the disease course; there was no statistically significant difference between patient groups. Consequences of patients’ criminal and socially inappropriate behavior included arrest, financial restrictions from authorities, dismissal from work, divorce, physical and emotional injuries, social isolation, and death.

In patients with FTD who exhibited criminal behavior, the expression of tau pathology was less common than the expression of non-tau pathologies (fused in sarcoma, transactive response DNA-binding protein 43, or undetermined owing to weak staining or lack of protein expression with applied stains), yielding a statistically significant difference (*P* < .001). An expression of non-tau pathology increased the odds for criminal behavior by a factor of 9.0 (95% CI, 3.4-24.0) (Table 3).

**Table 3.** Distribution of the Dominating Protein Pathology Among 119 Patients With Frontotemporal Dementia^a^

Protein Pathology	Total	No. (%) of Patients^b^	
		Criminal Behavior	Noncriminal Behavior
Tau	44	6 (13.6)	38 (86.4)
Non-tau	75	44 (58.7)	31 (41.3)
Fused in sarcoma	5	3 (60.0)	2 (40.0)
Transactive response DNA-binding protein 43	59	36 (61.0)	23 (39.0)
Undetermined^c^	11	5 (45.5)	6 (54.5)
Total	119	50 (42.0)	69 (58.0)

^a^ When comparing tau and non-tau pathology, we found a statistically significant difference in terms of criminal behavior. Of the 50 patients exhibiting criminal behavior, 44 (88.0%) had non-tau pathology (*P* < .001).

^b^ Data in the Total column provide the denominator for each row.

^c^ The protein pathology was undetermined owing to weak staining or a lack of protein expression with applied stains.

Many (although not all) of the patients with FTLD-tau had corticobasal degeneration/corticobasal syndromes and progressive supranuclear palsy. In the non-tau cases the pathology was predominantly severe in the frontal lobes, but sometimes also in the temporal lobes.

### Qualitative Results

#### Mismanagement of Personal Finances

As their disease progressed, several patients with AD experienced financial difficulties owing to forgetfulness when dealing with monthly bills. Patients with FTD appeared to spend considerable amounts of money on seemingly irrelevant items or activities and made several unrestrained financial transactions, including the purchase of real estate in bad condition and its subsequent sale at a substantial economic cost, reckless spending resulting in property loss, and blackmail of younger relatives in an attempt to cover financial debts.

#### Sexual Advances

Sexual advances by patients with FTD engaging in criminal behavior ranged from clear sexual abuses to sexual harassment and public masturbation. These behaviors were sometimes directed toward young children. Patients exhibiting inappropriate but not criminal sexual behavior made sexual advances to other patients.

#### Other

There were several patients in the both groups who vandalized their environment, including not only their own homes but, eventually, the nursing homes or hospital wards to which they were admitted. Some of them threatened others verbally, often quite severely. Criminal behavior in the “other” category further involved patients with FTD engaging in stalking or trespassing. Socially inappropriate behavior often included the invasion of other people’s personal spaces and aimless screaming, crying, or laughing. These behaviors sometimes resulted in physical injuries: when provoked, other patients reacted in a physically violent way. Several patients with FTD could, on request, verbalize that their criminal behavior was inappropriate. However, they continued to engage in it and find excuses for it.

## Discussion

Our study results suggest that criminal and socially inappropriate behavior are much more common in patients with FTD than in patients with AD. Our results regarding criminal behavior are in line with previous estimates.^4,5,6^ As opposed to previous studies on the topic, however, this study explored the prevalence of criminal behavior and socially inappropriate behavior throughout the entire course of disease, from onset to death, not just until the time of inquiry. This approach could explain the greater prevalence found. We have further found that patients with FTD had higher rates of recurrence of criminal behavior than patients with AD. This finding is not surprising because patients with FTD have often experienced damage to their frontal lobes, where impulse control (among other functions) is located.^10^

Numerous patients (30 of the 101 [29.7%] with AD and 21 of the 119 [17.6%] with FTD) urinated or defecated in inappropriate places, such as common areas in nursing homes (eg, living rooms, dining rooms, or other patients’ beds), but this behavior never reached criminal levels and was therefore deemed socially inappropriate behavior as opposed to criminal behavior. At any rate, patients’ tendency to urinate or defecate in nursing homes may be explained by the fact that their disease had likely progressed to a stage of incontinence and severe cognitive impairment by the time they were admitted into these homes.

It has previously been shown that patients with FTD interact more frequently with the police than patients with AD, most often owing to criminal behavior.^15^ The present study confirms this finding, although the present sample included 22 additional patients with FTD. We believe that the growing population of individuals with dementia will make our findings relevant to the police and society at large.

Several patients with FTD could verbalize that their criminal behavior was inappropriate. The ability to understand the criminal nature of an action and proceed regardless is problematic, especially when a patient with FTD is facing criminal charges.

The AD and FTD groups did not differ in terms of the time of onset of criminal behavior during the course of disease. A study from 2015 shows that criminal behavior may be the first sign of FTD.^5^ Other studies indicate that mild behavioral impairment can manifest long before other symptoms arise in patients with neurodegenerative diseases, especially FTD.^25^ The median age of the patients with AD in the present study was 64 years and might therefore not be representative of typical patients with AD, who have an older median age.^26^ The younger individuals would be expected to have more social, financial, and physical freedom, which in turn could make other people more likely to call attention to aberrant behaviors. If anything, a relative overrepresentation of patients with early-onset AD, as in the present study, would rather work to obscure the differences between the AD and FTD groups.

Although it is hard to state the exact year of symptom onset (eg, depression might be one of the first signs of dementia), we are confident with having sorted out the patients with psychiatric disorders only from the patients whose psychiatric symptoms manifested as an early symptom of dementia. The cohort has been clinically followed up closely for several years. The cohort is based on a neuropathologic diagnosis of FTD; hence, “phenocopies” of FTD, such as patients with primary psychiatric diagnoses who fulfill clinical FTD criteria but whose symptoms are unrelated to traditional neurodegenerative FTD, have been excluded.

To our knowledge, protein pathology in relation to criminal behavior has not previously been presented. We found that patients with FTD who committed crimes mostly exhibited non-tau pathology (transactive response DNA-binding protein, fused in sarcoma, or undetermined owing to weak protein expression staining), whereas patients with FTD but no criminal behavior mostly exhibited tau pathology. It appears that non-tau pathology may be associated with more severe behavioral problems that potentially lead to criminal behavior. This association between non-tau pathology and the higher prevalence of socially inappropriate and criminal behavior may be associated with the regional spread of brain damage rather than with the type of protein pathology in FTLD. Many (although not all) of the patients with FTLD-tau had corticobasal degeneration/corticobasal syndromes and progressive supranuclear palsy. Both conditions have a well-known preponderance of pathology in midline (including brainstem) structures, whereas the non-tau diseases, which to a great extent corresponded to the clinical syndromes semantic dementia/semantic variant of primary progressive aphasia/progressive nonfluent aphasia and to the behavioral variant of FTD, had much more of a cortical emphasis of the disease, being the locus of origin of behavioral control, empathy, and appropriate emotional responses.^10^

### Strengths and Limitations

This study’s strengths include the extensive clinical investigations conducted by specialists in geriatric psychiatry and cognitive medicine, the relatively long study period (51 years), and the neuropathologically verified, postmortem dementia diagnoses.

This study also had some limitations. First, because this was a retrospective medical records review, it was not possible to confirm or reject information about a particular patient’s behavior, since all patients had died. Moreover, findings were based on third-party interpretation of such behavior.

We were aware of neuropathologic diagnoses when reviewing the patients’ medical records and may hence have been subjected to bias when looking for criminal and socially inappropriate behavior. We cannot rule out the possibility that the physicians suspecting FTD might have investigated criminal behavior and socially inappropriate behavior more thoroughly at the time of annual visits. A prominent part of the clinical record notes, however, came from nursing homes and from family members who had themselves contacted the physician or the nurse to inform them about the odd behavior. Furthermore, we did not have access to criminal records and could therefore not search for more extensive details regarding patients’ criminal behavior. It is possible that the number of criminal incidents was higher because many patients or their families may not have wanted (or may have forgotten) to inform their physician about cases of criminal behavior. Our numbers on the prevalence of criminal behavior should therefore be deemed conservative. We believe this to be the case for our socially inappropriate behavior numbers as well.

When mapping criminal behavior, we decided to exclude physical aggression as a behavioral trait. First, as mentioned, a study of the same cohort has already been published on the matter.^7^ Second, physical aggression is prevalent among patients with a neurodegenerative disorder and is often exhibited when receiving intimate care. Many of the patient notes considered in this study were taken in nursing homes; therefore, a high number of notes regarding physical aggression was to be expected.

One might think that a specialized care center, such as the Memory Clinic (previously, the Psychogeriatric Department) in Lund, would only accept the most behaviorally disturbed or criminal patients and that this would lead to selection bias and skewed results.

Our interpretation of patients’ behavior and whether it should be considered criminal or socially inappropriate was partly drawn from subjective opinion, which in turn was based on our experience of life in Sweden and our knowledge of what is legal or deemed socially appropriate there. The 2 most challenging categories for us to decide on were mismanagement of personal finances and sexual advances. To aid in our evaluation of these matters, we consulted with a lawyer who specialized in mental health.

## Conclusions

This study’s findings suggest that criminal and socially inappropriate behaviors may be more prevalent among patients with FTD than among patients with AD. In addition, recurrence of criminal behavior during the course of disease may be more prevalent among patients with FTD than among patients with AD. Non-tau pathology was more prevalent than tau pathology among patients with FTD who exhibited criminal behavior. These findings may aid the clinical differential diagnostic process and the decision making process regarding patient care. We suggest that older individuals exhibiting criminal or socially inappropriate behavior for the first time be screened for neurodegenerative disorders. Prospective studies on this matter, including neuropathologic follow-up post mortem, are required.
